# Zebra Finch Introductory Notes Become Song-Like But Have Different Timing and Acoustic Correlations with the Song That Follows

**DOI:** 10.1523/ENEURO.0199-25.2026

**Published:** 2026-08-07

**Authors:** Divya Rao, Devika Babu Kizhakoot, Shikha Kalra, Harini Suri, Sonam Chorol, Raghav Rajan

**Affiliations:** Division of Biology, Indian Institute of Science Education and Research Pune (IISER Pune), Pune, Maharashtra 411008, India

**Keywords:** introductory notes, motor preparation, naturally learned movements, song initiation, songbird, zebra finch

## Abstract

Male zebra finches begin their song bouts by repeating a short sound called an introductory note (IN). As INs repeat, their timing and acoustic properties change, similar to premovement neural preparatory activity seen in primates and rodents. Hence, INs are thought to reflect motor preparation for starting song. However, INs themselves are vocalizations/movements, like the song that follows, suggesting that INs could be part of the song sequence. To determine how similar songs and INs are, we compared their properties in adult, male zebra finches recorded across 3 years. First, song and INs simultaneously changed in the first year post-hatch; as described earlier, songs became faster and we found an increase of 0.58 ± 0.07 in mean IN number. Second, as described earlier, IN properties changed from first to last IN. Here, we found that these changes were driven by the last IN becoming more like the first song syllable in duration, amplitude, and timing. Third, within each bout, first song syllable timing was correlated with last IN timing in only 18% of birds. Amplitude and mean frequency of the first song syllable was correlated not only with the last IN, but also with the first IN and second song syllable in >40% of birds. Finally, the reduction in gaps between successive INs was not seen in other types of repeat syllables. Overall, the acoustic similarities between INs and song suggest that INs are part of the song sequence, but timing differences suggest different mechanisms for controlling IN timing.

## Significance Statement

Simple movements are preceded by neural preparatory activity before execution, and recent studies have suggested that the complex song sequence of the zebra finch is also preceded by preparatory vocalizations called introductory notes (INs). However, given that INs are also vocalizations, it remains unclear whether their properties reflect similarities to song. Here we found that as INs progress to song, both timing and acoustic properties of INs became more similar to song. In contrast, within bout correlations between INs and song were more prevalent for acoustic features and not timing. These results highlight similarities between INs and song, while also suggesting potential differences in the control of IN timing and acoustic properties.

## Introduction

**Figure 1.**
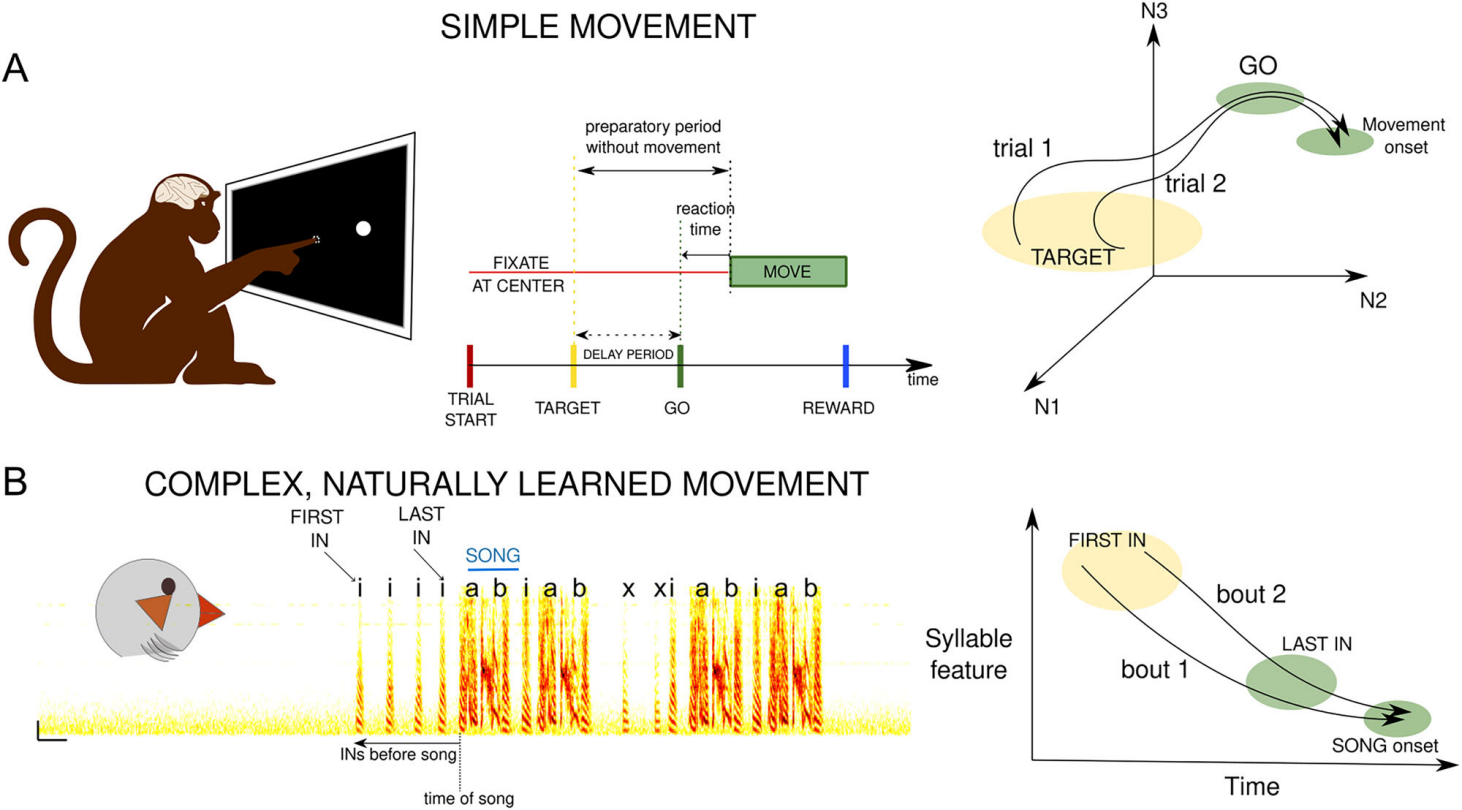
Preparatory activity and its relationship to upcoming movement. ***A***, Simple movement in monkeys: movement of finger from a central fixation point to a target as part of delayed reaching tasks (left). Structure of the task with the different elements of the task shown in the middle. Right, The neural activity on each trial (represented by the black traces), during the preparatory period, converges on a consistent point before movement onset. ***B***, Zebra finch song is an example of a complex, naturally learned movement. “*i*”s represent introductory notes (INs), “ab” represent song sequence. Properties of INs converge on a consistent state before the onset of the first song syllable “*a*”.

Simple movements, like reaching for an object, are preceded by a “preparatory” period (Fig. 1*A*, left) when neural activity progresses from a variable initial state to a consistent final state just before movement initiation (Fig. 1*A*, right; Churchland et al., 2006b, 2010; Shenoy et al., 2011; Li et al., 2016; Svoboda and Li, 2018). Recent studies suggest that preparation also precedes complex, naturally learned, movement sequences, like the well-studied song of the adult male zebra finch (Rajan and Doupe, 2013; Rao et al., 2019), but the nature of this preparation remains poorly understood.

Zebra finch song is a stereotyped sequence of sounds interleaved by silent gaps (Fig. 1*B*, left, “*a*” and “*b*” represent the song sequence). It is part of the courtship ritual of the male zebra finch (Sossinka and Böhner, 1980; Zann, 1996; Fee and Scharff, 2010) and is naturally learned by young birds (Immelmann, 1969; Price, 1979; Zann, 1996). Song bouts begin with variable repetitions of a short sound called an introductory note (IN, marked as “*i*” in Fig. 1*B*) (Price, 1979; Sossinka and Böhner, 1980). As INs repeat, their timing and acoustic features change; gaps between successive INs reduce and become more stereotyped, and acoustic features reach a consistent last IN just before the start of the first song (Fig. 1*B*, right; Rajan and Doupe, 2013; Rao et al., 2019). This progression resembles neural preparatory activity going from a variable, initial state to a consistent, final state before movement initiation. Neural preparatory activity has been extensively characterized in rodent and primate brains and is believed to represent motor preparation for initiation of simple movements (Shenoy et al., 2011, 2013; Churchland, 2015; Svoboda and Li, 2018). By analogy, INs might also reflect motor preparation for complex, naturally learned song sequences.

In addition to similarities in progression to movement, INs and neural preparatory activity both correlate with features of the upcoming movement. In primates, premovement neural activity correlates with upcoming movement parameters on a trial-by-trial basis (Churchland et al., 2006a). Similar correlations exist in zebra finches, where similar last INs are followed by similar first song syllables (Rajan and Doupe, 2013). This adds support to the hypothesis that INs reflect motor preparation for initiating the complex song sequence.

Despite these similarities, one important difference remains. Preparatory neural activity occurs before the movement and is itself not accompanied by movement. In contrast, INs are movements (vocalizations) like the song that follows. Additionally, neurons in multiple song system nuclei, in the zebra finch brain, show activity changes before the first IN begins, while the bird is quiet (Hessler and Doupe, 1999; Kao et al., 2008; Woolley et al., 2014; Rajan, 2018; Daliparthi et al., 2019). Recent studies suggest this activity reflects preparation for song initiation (Rajan, 2018; Daliparthi et al., 2019). Then what do INs represent? INs could reflect either a “vocalized” continuation of preparatory activity or they could be variable vocalizations that are part of the song sequence, similar to variability seen in the song sequences of related songbird species, like the Bengalese finch (Okanoya, 2004). To address these possibilities, we recorded and analyzed INs and songs from multiple adult, male, zebra finches over a 3 year period.

Specifically, we first characterized age-related changes in INs. Song is known to speed up and become more stereotyped over the first year of age (Brainard and Doupe, 2001; Pytte et al., 2007; Glaze and Troyer, 2013; James and Sakata, 2019). If INs are part of the song sequence, IN features should also show similar age-related changes. Previous studies have shown that IN features do not change over a 5 d period (Rao et al., 2019), but longer term changes remain unclear. Here, we characterized IN changes over the first 3 years post-hatch through repeated recordings of individual birds.

Second, we compared IN features with first song syllable features. While IN timing and acoustic properties change from first to last IN and similar last INs precede similar first song syllables (Rajan and Doupe, 2013), what features drive these changes and whether they are in the direction of song remains unclear. The neural preparatory hypothesis does not make specific claims about the direction of change; preparatory changes in activity set the initial state for movement dynamics to unfold without necessarily being in the direction of movement related activity (Churchland et al., 2010; Shenoy et al., 2011, 2013). However, if INs are part of the song sequence, one would expect IN features to change and become closer to song features. Additionally, one would also expect similar correlations between the first IN and the first song syllable and between the first and second song syllables. The presence of such correlations would suggest the existence of global correlations between vocalizations within a song bout and be consistent with the idea of INs being part of the song sequence.

Finally, INs involve repeating the same syllable unlike typical zebra finch songs that consist of distinct, nonrepeating syllables. However, songs of a small fraction of zebra finches do contain repeats of song syllables and nonsong vocalizations (Helekar et al., 2003). Therefore, we also compared IN properties with those of other repeating syllables to determine whether IN properties reflect general properties of repeating syllables.

## Materials and Methods

All experiments at IISER Pune were approved by the Institute Animal Ethical Committee in accordance with the guidelines of the Committee for the Control and Supervision of Experiments on Animals, New Delhi. All song recording procedures for songs recorded at UCSF were approved by the UCSF Institutional Animal Care and Use Committee in accordance with NIH guidelines. We recorded songs from a total of 54 adult male birds (age >90 d post-hatch) with some purchased from an outside local vendor (*n* = 24) and some bred at IISER Pune (*n* = 24) or UCSF (*n* = 6). The age of all purchased birds was assumed to be 60 d post-hatch (dph) as they had red beaks (beaks are black for birds <60 dph). At times when birds were not being recorded, they were housed in large cages with 5–8 other birds in a bird colony maintained with a 14/10 h light/dark cycle. Food and water were provided at all times without restrictions.

### Song recording

Birds were isolated from the colony and placed in separate cages (cube of side 9 inches) in a sound attenuation enclosure (NewTech Acoustic Systems) maintained with a 14/10 h lightdark cycle. A microphone (AKG Acoustics C417PP in IISER Pune or B3 omnidirectional lavalier microphone, Countryman Associates in UCSF) was clipped on the roof of the cage to record song. Signals from the microphone were amplified (Behringer XENYX 802 in IISER Pune or Krohn-Hite preamplifier in UCSF) and then digitized on a computer.

Songs were recorded either in “triggered” or “continuous” mode. Briefly, in “triggered” mode, periods of recordings that crossed a preset threshold were saved along with an additional 1–3 s of data flanking this period on either side. In “continuous” mode, audio files were saved continuously for the entire recording period. All data were saved to disk at a sampling rate of 44,100 Hz (for 7 birds, there were a few days recorded with a different software that saved data at 32,000 Hz) using custom-written software (Python or C). The different sets of birds used for different analyses and the overlap between these sets are explained below.

### Birds used for analysis of age-related changes and IN–song correlations

**Figure 2.**
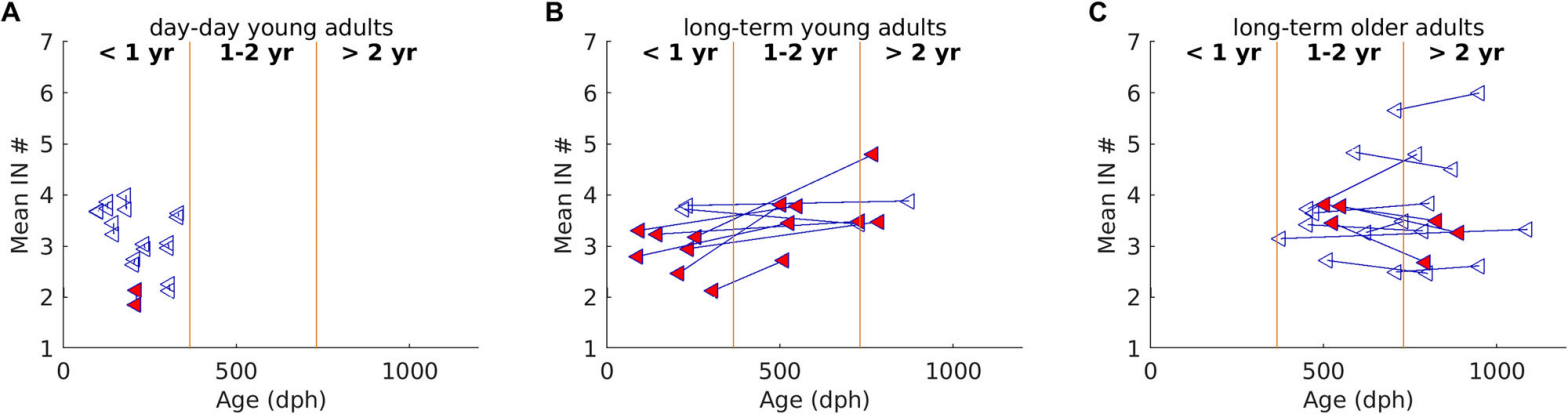
IN number increases in the first year of age. ***A–C***, Blue lines joining triangles represent mean IN number for selected pair of days for each bird, within each of the three groups. (1) Day–day change in young adults: two days <5 d apart (day–day) and both days <1 year post-hatch (***A***). (2) Long-term change in young adults: two days >5 d apart with first day <1 year post-hatch and second day >1 year post-hatch (***B***). (3) Long-term change in old adults: two days >5 d apart (long-term) with both days >1 year post-hatch (***C***). Pairs of days, in each bird, were chosen such that there was maximum difference in mean IN number between those two days. In ***A–C***, filled red triangles indicate significant differences (*p* < 0.05) between days and unfilled blue triangles indicate non-significant differences between days (*p* > 0.05). Results of statistics in Extended Data Figure 2-1.

**Figure 3.**
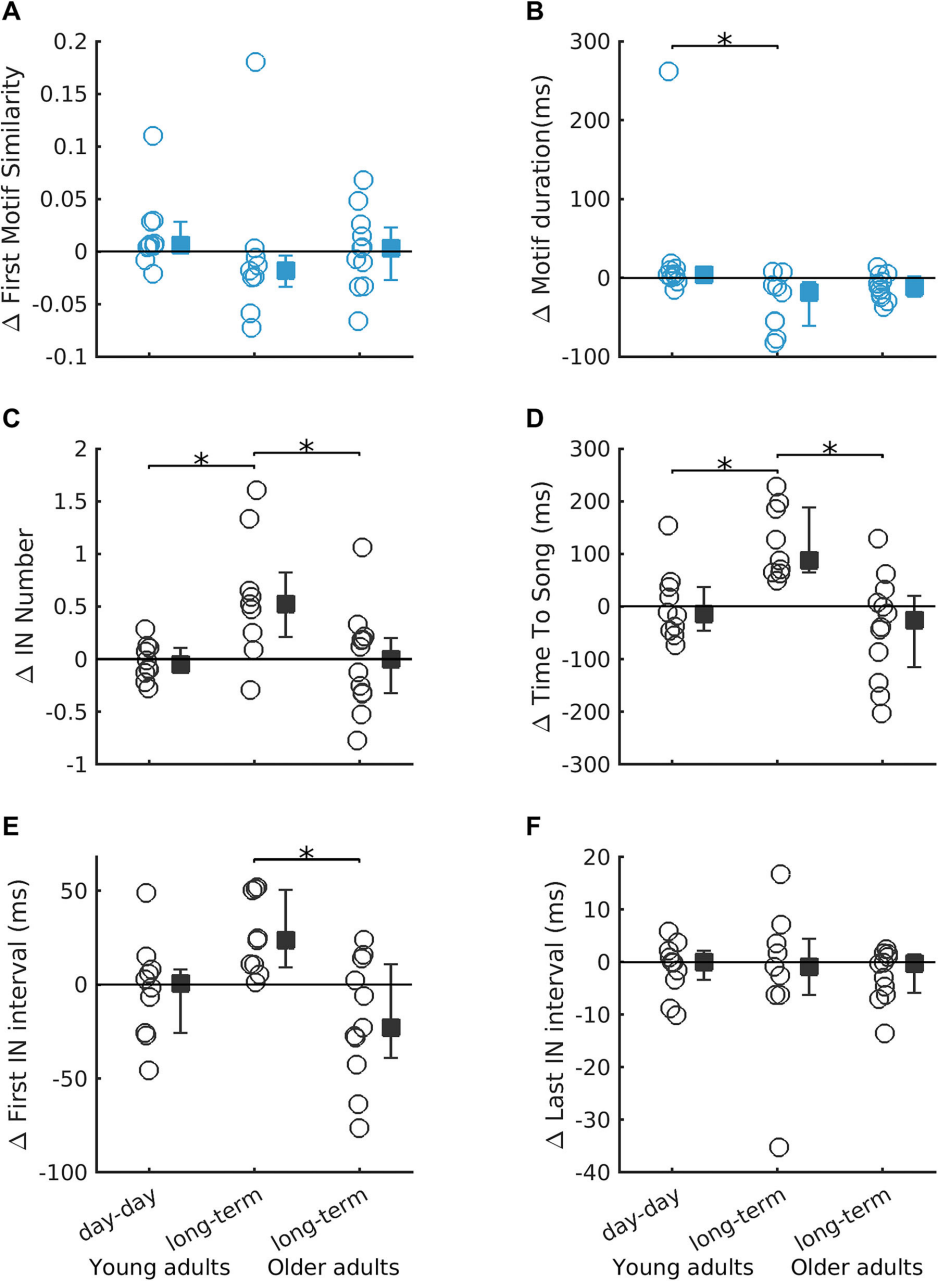
Song and IN properties simultaneously change in the first year of age. ***A–F***, Changes in motif similarity (***A***), motif duration (***B***), IN number (***C***) time-to-song (***D***), first IN interval (***E***), and last IN interval (***F***) are plotted across different age categories. Circles represent change in mean property between pairs of days in a particular age category for individual birds. Square and whiskers represent median and interquartile range for the three age groups. *denotes *p* ≤ 0.05 Kruskal–Wallis ANOVA followed by Tukey–Kramer test. See additional data in Extended Data Figure 3-1 and results of statistics in Extended Data Figure 2-1.

**Figure 4.**
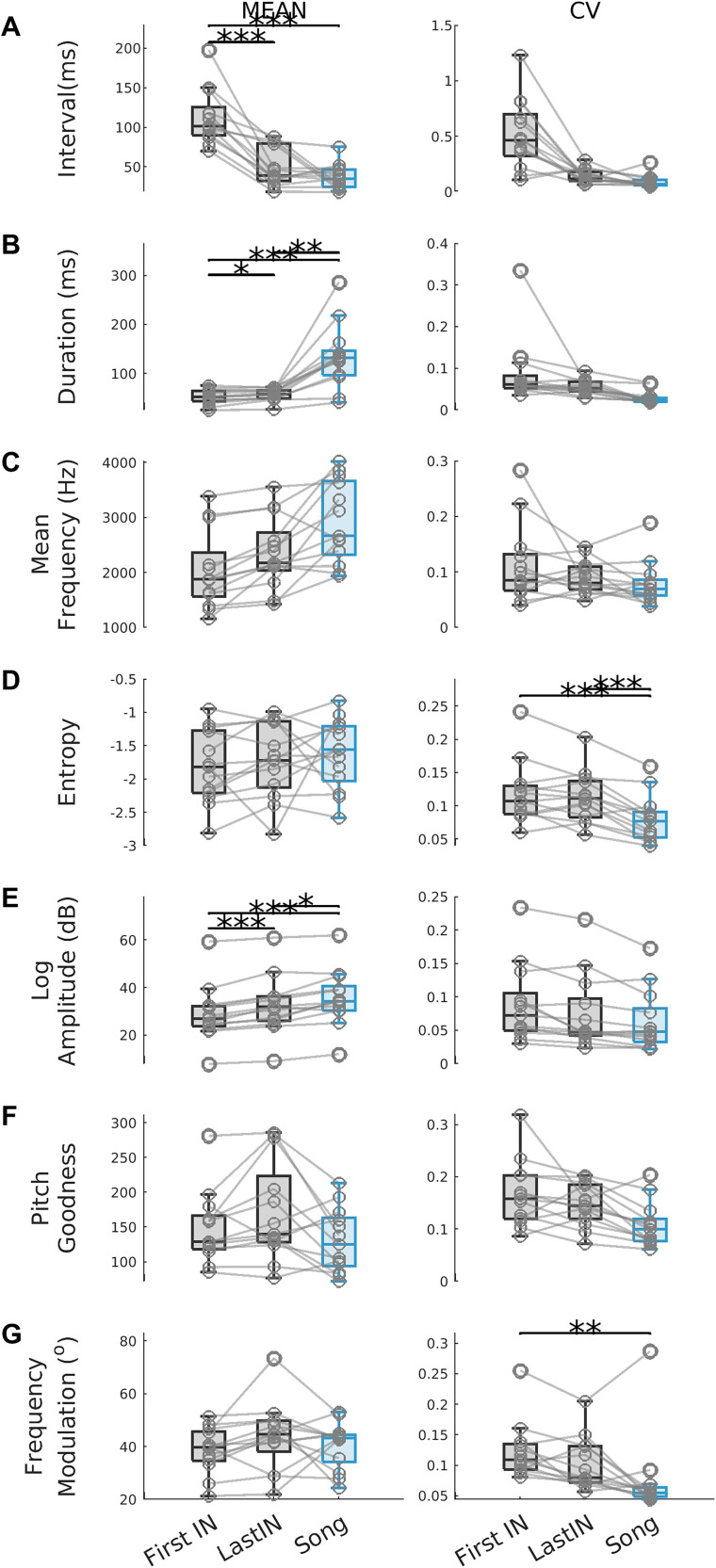
Timing and acoustic properties of introductory notes (IN) change in the direction of song. ***A–G***, Comparison of properties between First and Last IN position (gray) and the first syllable of the upcoming song (blue). Circles joined by lines represent data from individual birds, and boxplots represent group data across birds. Column 1 shows mean and column 2 shows CV or coefficient of variation for intervals (***A***), syllable acoustic properties namely duration (***B***), mean frequency (***C***), entropy (***D***), log amplitude (***E***), pitch goodness (***F***), and frequency modulation (***G***), respectively. * denotes *p* ≤ 0.05, **p ≤ 0.01, ***p ≤ 0.005, repeated-measures one-way ANOVA followed by post hoc Tukey–Kramer test. See results of statistics in Extended Data Figure 2-1.

**Figure 5.**
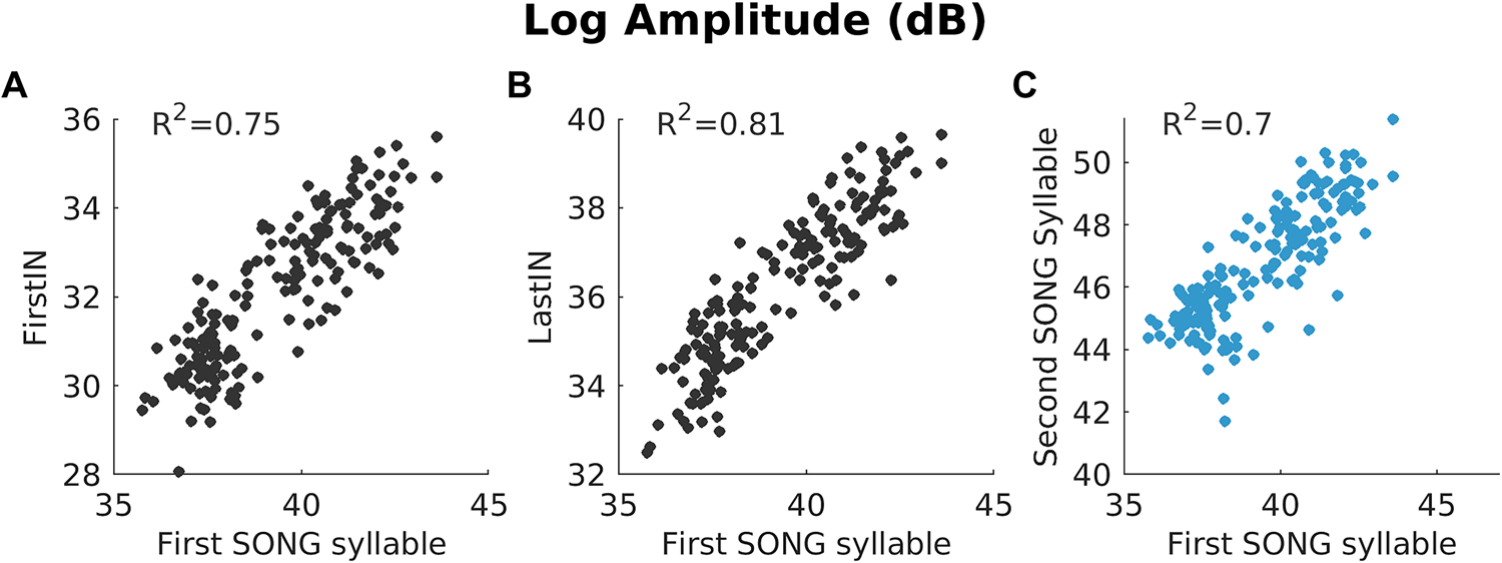
Representative example of within bout correlations between acoustic properties of IN and song. ***A–C***, Example session from one bird that shows significant positive correlations for log amplitude of first song syllable in the (first) motif along *x*-axis with (***A***) first IN position (black circles), (***B***) last IN position (black circles), and (***C***) second song syllable (blue circles), respectively, along *y*-axis. Circles represent data from individual bouts. *p* ≤ 0.05, Pearson’s correlation coefficient calculated after removing outliers. Results of statistics in Extended Data Figure 2-1.

**Figure 6.**
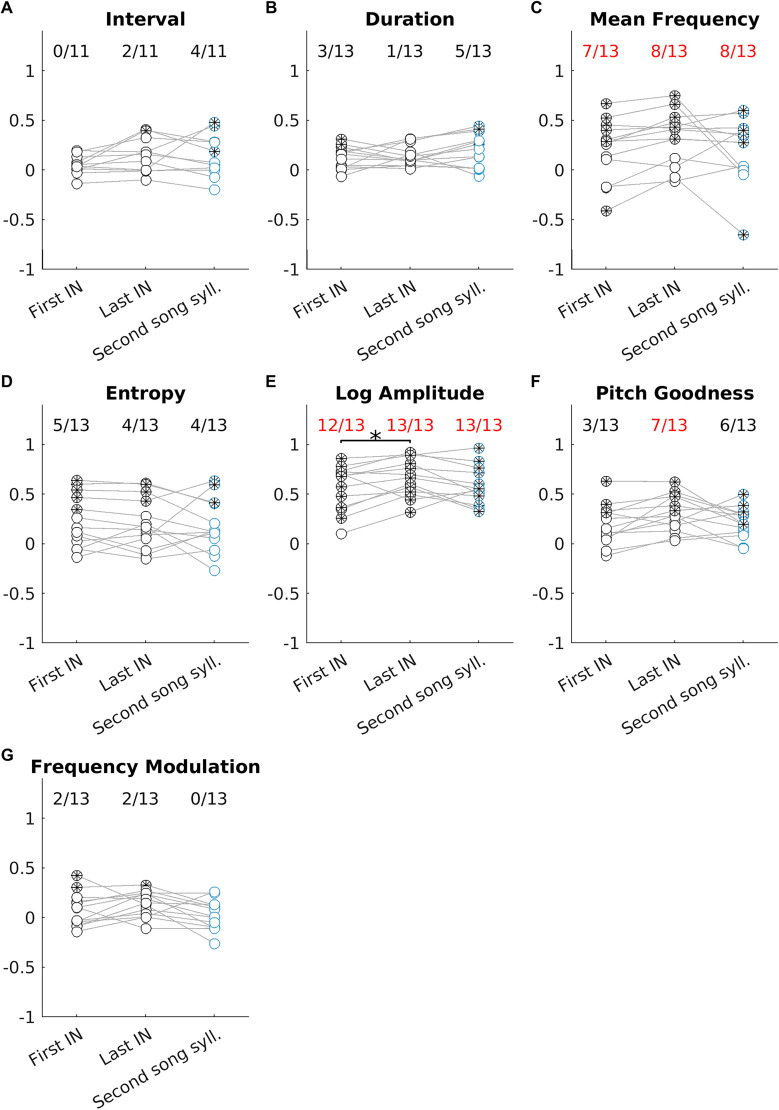
Correlations with first song syllable. IN timing and acoustic properties (duration, mean frequency, entropy, log amplitude, pitch goodness, and frequency modulation for a syllable) for the first IN, last IN, and second song syllable were correlated with corresponding properties of the first song syllable across bouts. Each circle represents *r* values for within bout correlations (Pearson’s correlation) in one bird. Lines connecting circles represent data from the same bird. Circles with a “*” represent significant correlations and unfilled circles represent non-significant correlations. Proportion of birds with significant correlations are indicated on top and marked with red if proportions were ≥0.5. * denotes *p* ≤ 0.05, ** denotes *p* ≤ 0.01, Wilcoxon signed-rank test. See additional data in Extended Data Figures 6-1, 6-2 and results of statistics in Extended Data Figure 2-1.

Twenty birds were recorded on multiple days (median 3 d per bird: range 2–8 d per bird) in the age range from 89 to 1,087 dph. As far as possible, we maintained the same position of the microphone for a given bird on different days of recording. A subset of birds and days (2 d each from 14/20 birds) overlapped with those analyzed for day-to-day changes in IN properties in a previous study. One bird (1/20) overlapped with the birds used for ts-cut surgery in that same study, but the recording days analyzed here are different and are from well before the surgical procedure. A subset of birds (5/20) were recorded earlier at UCSF and had recordings from 2 nearby days, <5 d apart, included in this analysis. For characterizing IN changes (Fig. 4) and within bout correlations of INs and song (Figs. 5, 6; Extended Data Figs. 6-1, 6-2), songs recorded from 1 d (<1 year of age) in each bird were analyzed. Multiple sessions were analyzed for age-related changes in INs and songs (Figs. 2, 3). The difference between days of recording spanned a wide range (1–812 d) and the time of recording varied across days for the same bird.

### Data overlap with previous studies

Songs of birds from UCSF were recorded as part of a different study for characterizing INs. Besides characterizing the properties of INs in every sequence and the associated neural activity during INs, the study also correlated the similarity of pairs of last INs to pairs of first song syllables. However, the direct relationship between individual properties of INs and songs was not compared. Here, we used these songs to directly compare the properties of INs and songs.

The IN–song correlation analysis was repeated for one session of undirected song, each, from seven birds with head-implanted microphones, five of which were recorded earlier for a different study (Suri and Rajan, 2018). The distance between the bird and the microphone does not change in head-implanted microphone recordings. Hence, they were used to control for amplitude measurement differences that may arise due to changes in relative position of the bird and the microphone in suspended microphone recordings. IN–song correlations were not analyzed as part of the original study.

IN–song correlation analysis was repeated for one session of undirected song, each, from seven birds with a cannula inserted into their air-sacs for respiratory pressure recordings as part of another study (Kizhakoot et al., 2025). Respiratory pressure is correlated with song amplitude (Plummer and Goller, 2008; Zollinger et al., 2011) and is a measure of amplitude that is not influenced by the nature of the recording environment or the distance between the microphone and the bird. IN–song correlations were not analyzed as part of the original study.

IN–song correlation analysis was repeated for one session of undirected song, each, from six birds recorded after ts-nerve (tracheosyringeal nerve) cut surgery as part of an earlier study (Rao et al., 2019). This data provided an advantage to look at IN–song acoustic correlations in the absence of properties related to syllable identity as these are lost following ts-nerve cut manipulation. IN–song correlations were not analyzed as part of the original study.

### Data for repeat syllables

Songs of 17 birds were identified that specifically repeated at least one syllable within the motif. One session each from these birds was used to compare the properties of different types of repeated syllables produced by the bird, namely, INs, motif syllable repeats, and calls. One session from one bird overlapped with the set used for IN–song correlations. One of the birds overlapped with ts-nerve cut birds and pre-ts-nerve cut songs were analyzed for repeat syllable properties. One of the birds recorded in this set was also bred and recorded earlier at UCSF.

### Data analysis

All the analyses were performed using custom-written scripts in MATLAB (www.mathworks.com). Audio files were processed and vocalizations were labeled using procedures described earlier (Rao et al., 2019). Briefly, audio files were segmented into syllables based on a user-defined amplitude threshold. Consecutive syllables with intersyllable gaps shorter than 5 ms were merged and syllables shorter than 10 ms were deleted. Labels were assigned to syllables in a semi-automatic manner. Automatic labels were assigned using a modified template-matching procedure (Glaze and Troyer, 2006) or KlustaKwik clustering (http://klustakwik.sourceforge.net/) of acoustic features calculated using Sound Analysis Pro (https://soundanalysispro.com/matlab-sat). Labels were then manually checked for all files. Files were split into bouts and all bouts containing songs (or motifs, used interchangeably) were selected for analysis. Typically these bouts consisted of repeating IN sequences before the first song.

#### Defining a bout and bout interval criteria

A bout was defined as a period of vocalizations separated by 2 s of silence (Sossinka and Böhner, 1980). This criteria was applied to select bouts from recordings in “continuous” mode. However, many audio files recorded in “triggered” mode did not have 2 s of silence before the first syllable in the file. This occurred when the initial vocalizations in the bout were soft and the set trigger threshold was crossed by a vocalization that occurred later in the bout. Such audio files had <2 s of silence before the first syllable in the file. However, we assumed that there was silence before the start of the file as the set trigger threshold was not crossed and so we included such files in our analysis. The bout criteria for triggered recordings was reduced to include enough audio files (>15 bouts) for analysis and the criteria ranged from 0.5 to 1.5 s only for the beginning of the file. Within a file, we still considered 2 s of silence and we always considered 2 s of silence for continuous recordings. For a given bird, we also maintained the same bout criteria across days. This was important as we observed that mean IN number, at the beginning of the bout, depended on bout interval criteria; as shown previously, mean IN number reduced a little when considering a bout criteria of <2 s (Sossinka and Böhner, 1980; Rajan and Doupe, 2013). However, mean IN number was not different between days when the same bout interval criteria was applied.

#### Determining minimum number of bouts for IN analysis

The number of INs varied across bouts and the number of bouts sung by birds varied across days and birds (median 73.5 song bouts per day: range 4–430). It was important to characterize how the number of bouts affected the estimate of mean IN number for a day and to determine the minimum number of bouts beyond which this estimate did not change considerably. For all days with >100 song bouts (30 d), a range of different number of bouts were subsampled randomly and the corresponding average standard deviation across 1,000 iterations of Monte Carlo simulations was calculated. The minimum number of bouts for IN analysis was identified as the number of bouts beyond which increasing the number of bouts did not change the average standard deviation by >0.01. The standard deviation met our criteria at 10 bouts for 18/30 sessions and at 15 bouts for 28/30 sessions. Hence, across all days and birds, only sessions with at least 15 song bouts were selected for IN analysis. Based on this criteria, we excluded two days from one bird, each with <15 song bouts.

#### Calculating IN and song properties

Unless otherwise specified, we considered only INs at the beginning of the bout and the first motif that followed immediately after the INs, for analyzing IN and song properties across bouts. If a call (non-song vocalization) preceded INs at the beginning of the bout, then the bout was considered only if the call occurred >200 ms before first IN. This was based on previous work that showed that the number of INs was reduced, when calls preceded the first IN by <200 ms (Rao et al., 2019). For each song bout, the number of INs was calculated preceding the first motif. The timing and acoustic properties of INs and song were calculated as follows: timing of IN for first position or first gap was the silent time interval between offset of first IN and onset of second IN. Timing of IN for last position or the last gap was calculated as the silent time interval between offset of last IN and the onset of the first song syllable. Song timing for the first position in the motif was calculated as the silent time gap between offset of first motif syllable and onset of second motif syllable. Song timing for the second position in the motif was calculated as the silent time gap between offset of second motif syllable and onset of third motif syllable. Similar to timing, the acoustic properties of INs were calculated for first and last IN and for the first and second motif syllable. For comparisons of timing and acoustic properties involving changes in IN from first to last position, birds with two types of INs in their IN sequences were excluded as the syllable identity in these birds would be different for first and last IN. For comparing INs and the first song syllable (Fig. 4), bouts with at least two INs were used, such that each bout selected had a first IN, a last IN and a first song syllable. For measuring within bout correlations in acoustic properties (Figs. 5, 6; Extended Data Fig. 6-2), bouts with at least two INs and a song with at least two syllables were used to ensure that each selected bout had a first IN, a last IN, a first song syllable, and a second song syllable. For measuring within bout correlations in timing (Figs. 5, 6; Extended Data Fig. 6-2), we used bouts with at least two INs and a song with at least three syllables, so as to include all of the gaps mentioned above.

#### Acoustic properties of syllables

The acoustic properties for IN and song syllables were measured as described by Sound Analysis Pro 2011 (Tchernichovski et al., 2000; http://soundanalysispro.com/) using SAP matlab code (https://soundanalysispro.com/matlab-sat). These basic features reduced the dimensionality of the complex sound spectrogram for analysis and made it easier to understand changes in different aspects of sound. A brief intuitive understanding of the features is described below:

1. Log amplitude: It measures the loudness of a syllable. The intensity or power in the audio signal is measured relative to an arbitrary baseline for silence. It is reported in log scale units or dB.
2. Duration: Time from onset to offset of the syllable. Onset and offset are determined based on an amplitude threshold that distinguishes sound from silence in the audio signal. It is measured in units of seconds or milliseconds.
3. Mean frequency: The audio signal can be decomposed into different frequencies present in the signal. The mean frequency is a pitch measure that assesses the center of the distribution of intensity across different frequencies. It is measured in Hz.
4. Pitch goodness: It measures the periodicity of harmonic pitch (frequency stacks) in the syllable. Syllables with harmonics or “frequency stacks” (as observed in a spectrogram) have higher value, and syllables that are noisy or pure tone have lower value.
5. Wiener entropy or entropy: It measures the noisiness in the syllable. A noisy syllable appears as broadband or white noise in the spectrogram with intensity spread equally across all frequencies. White noise has an entropy value of 1 and pure tone (sound intensity concentrated at one frequency) has a value of 0. However, this is converted to a log scale of 0 to minus infinity. More noisy syllables will have entropy 0.
6. Frequency modulation: It is an estimate of the slope of the frequency trace on the spectrogram, measured in degrees. The steeper the slope, the higher the modulation.

#### Analyzing age-related changes in IN and song properties

Age-related changes were calculated as changes in mean or variability of properties between pairs of days belonging to either of the three age groups: (1) short-term changes in young adults, i.e., difference between pairs of days <5 d apart for young adults (both days were <1 year of age); (2) long-term changes in young birds, i.e., difference between pairs of days with one day <1 year and the other day >1 year; and (3) long-term changes in old adults, i.e., differences between pairs of days >5 d apart in old adults (both days >1 year old). If a bird was recorded for >1 d within an age group, we compared all pairs of days with each other and for each age group category, we chose pairs of days with the maximum change in IN number (Kruskal–Wallis test for all days followed by post hoc Tukey–Kramer test for comparing pairs of days). We reasoned that this would give us the maximum chance of detecting changes, if there were any. The same pairs of days were used to calculate age-related changes in other IN and song properties to understand the extent of age-related changes in other properties when IN number changes were maximum.

Changes in IN and song properties between two different days were measured by subtracting the mean or variability calculated for the earlier day from those measured on the later day. We calculated changes in mean IN number, IN timing, IN acoustic properties, and time-to-song. Time-to-song was defined as the duration of the IN sequence from onset of the first IN to onset of the first song syllable. To quantify song changes with age, parameters of song that have earlier been reported to change with age were calculated. These included motif duration and song similarity (Brainard and Doupe, 2001; Pytte et al., 2007; Glaze and Troyer, 2013; James and Sakata, 2019). Motif duration was measured as the time from the onset of the first motif syllable to the offset of the last motif syllable. Song similarity was calculated as average similarity index between pairs of motifs from a recording day. Ten motifs of a fixed syllable sequence were randomly selected across all motifs, and each of the 10 motifs was compared with the nine other motifs using a similarity index algorithm (Mandelblat-Cerf and Fee, 2014), followed by taking the mean for all pairs. The similarity index gave a measure of how similar songs on a given day were to each other. Similar to other properties, changes between days was measured by subtracting the similarity index of an earlier day from a later day.

Change in other song properties related to song sequence and individual timing and acoustic features of first song syllable were also measured between days. To compare changes in song similarity between pairs of days, we measured the difference between the average similarity indices of 10 randomly selected motifs on the two days. To compare song temporal structure between days, the temporal pattern of the motif sequence was obtained by taking the amplitude profile and replacing this with “1 s” for the duration of syllables and “0 s” for the duration of intervals between syllables. Two patterns, corresponding to the two motif sequences to be compared, were compared up to the duration of the shorter sequence using cross-correlation. The change between pairs of days was measured by subtracting the values of an earlier day from a later day.

#### Analysis of respiratory pressure recordings

Respiratory pressure recordings were analyzed as described previously (Kizhakoot et al., 2025). Briefly, baseline respiration for each file was calculated using one to three silent periods, each 3 s in duration. From each silent period, the middle 1 s segment was extracted, and respiratory pressure values from all such segments were averaged to determine the baseline respiration level. The first data point in a stretch of data points exceeding this baseline was defined as expiration onset, and the first data point in a stretch of data points falling below the baseline was defined as expiration offset. For every syllable, we calculated the mean expiratory pressure as the average of all data points between expiration onset and expiration offset. Expiratory mean amplitude (a measure of syllable amplitude) was calculated by subtracting the baseline respiration from the expiratory mean.

#### Analysis of repeat syllables

Repeat syllables belonging to either motifs, INs or calls, were analyzed for each bird. We considered only IN bouts where the bout started with an IN. Call repeats outside of song were considered as these were more common in all the birds we analyzed. While IN repeats proceed to just one song syllable type, other types of repeats were sometimes followed by different types of syllables in different bouts. To remain uniform with IN repeats, for other types of repeats, we considered the most probable “next” syllable after the repeat and analyzed only repeat instances that proceeded to this syllable. Similar to criteria used for INs in other datasets, repeat syllables with more than 15 instances were analyzed. The number, gap and acoustic properties were analyzed using a similar procedure as that described for INs above. We also calculated ratios of features to quantify how the repeat sequence proceeds. For gaps and acoustic properties, the ratio was defined as the gap or acoustic property at the current position divided by the corresponding gap or acoustic property at the next position in the sequence. A ratio of 1 indicated that feature values (gaps or acoustic properties) did not change over the repeat sequence, a ratio <1 indicated a decrease in feature values over the repeat sequence and a ratio >1 indicated an increase in feature values. For each bird and each feature, a median ratio was calculated across bouts.

### Experimental design and statistical analysis

All groups tested for differences had comparable sample numbers. For all comparison of independent groups of unequal sample sizes, we used nonparametric Kruskal–Wallis test. If the *p* value was ≤0.05, Tukey–Kramer's post hoc test was used to identify pairs of groups that were significantly different. These group comparisons included age-related changes in mean IN or song properties and comparison of mean properties of repeat syllables belonging to either motifs, INs or calls. Differences between (more than two) bird-matched groups were tested using repeated-measures one-way ANOVA. If the *p* value was ≤0.05, Tukey–Kramer's post hoc test was used to identify pairs of groups that were significantly different. This included comparison of mean or CV of syllable properties across bouts based on position, i.e., first IN, last IN, and first song syllable. Paired-group comparisons were tested for differences using Wilcoxon signed-rank test with a *p* value criteria of 0.05 for significant differences. These comparisons included significant correlation coefficient estimates of first IN–first song syllable with that of last IN–first song syllable and comparisons of first and last position gaps for repeating syllables. All within bout correlations were measured using Pearson's correlation coefficient of corresponding IN and song property values across bouts. Prior to measuring correlations, the outliers were removed (percentage removed: median, 2.41% data points; range, 0–41.03%). Outliers were detected as values beyond three times the median absolute deviation from the median. The correlations were calculated only if the number of paired sample points were >10 and were considered significant if *p* values were ≤0.05. The range of *R*-squared values for significant correlations were reported across birds along with proportion of birds with significant correlations. The statistical tests and results for all analyses are listed in Extended Data Figure 2-1.

### Data and code accessibility

All data and scripts for analysis are available on request from the corresponding author.

## Results

### Mean IN number before song increased by a small amount in the first year of age

To characterize changes in IN properties with age, we recorded individual birds on multiple days (*n* = 20 birds; median number of days per bird, 3; range per bird, 2–8 d) from ∼90 d post-hatch to ∼3 years of age. As mentioned earlier, song changes with age (Brainard and Doupe, 2001; Pytte et al., 2007; Glaze and Troyer, 2013; James and Sakata, 2019), and we expected INs to show similar age-related changes, if INs are also part of the song sequence. To characterize the extent of changes in IN properties with age, we considered three categories, namely, (1) short-term changes in young birds, i.e., difference between pairs of days <5 d apart for young adults (both days were <1 year of age); (2) long-term changes in young adults, i.e., difference between pairs of days with one day <1 year and the other day >1 year; and (3) long-term changes in old adults, i.e., differences between pairs of days >5 d apart in old adults (both days >1 year old). For each bird and category, we identified recording days that fell in this category and calculated the maximum difference in IN number to characterize the maximum extent of change (Fig. 2*A*–*C* represents days chosen for each of the three categories, respectively; see Materials and Methods for details). Significant changes were present mainly in the 2nd category (Fig. 2*B* has more red triangles indicating significant changes in mean IN number between days). These results show significant changes in mean IN number in the first year post-hatch.

**Figure 7.**
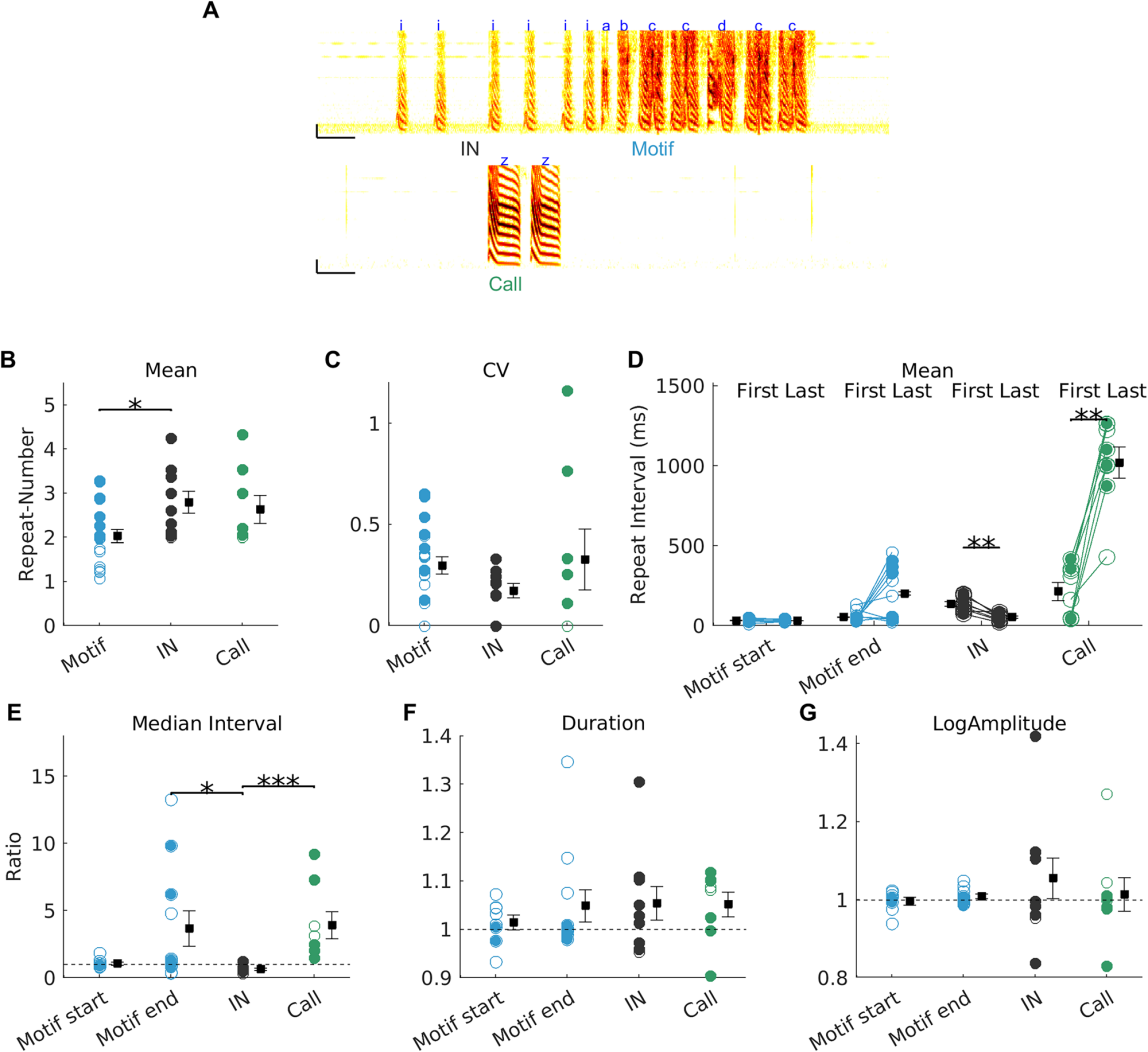
Speeding up of intervals between successive INs is not present in other types of repeats. ***A***, Top, Example spectrogram of a bird that produces IN repeats (represented by “*i*”) and song syllable repeats (represented by “*c*”). Bottom, Example of a bird producing call syllable repeats (represented by “*z*”) outside song. ***B***, ***C***, Comparison of mean repeat number (***B***) and repeat number variability (***C***) for the three types of repeats. ***D***, Comparison of “first” interval between successive syllables within the repeat and “last” interval between last syllable within the repeat and the next syllable for all three types of repeats. ***E–G***, Comparison of median ratio of repeat interval (***E***), mean ratio of duration (***F***), and log amplitude (***G***) between successive syllables within the repeat across different types of repeats. Circles represent data from individual birds, squares, and whiskers represent the mean and SEM across birds. In ***D***, lines join data from the same repeat syllable across different positions. In ***B***, * denotes *p* < 0.05, Kruskal–Wallis ANOVA. In ***D***, ** denotes *p* < 0.01 Wilcoxon signed-rank test. In ***E***, * denotes *p* < 0.05, *** *p* denotes <0.005, Kruskal–Wallis test, followed by post hoc Tukey–Kramer test. Dashed lines in ***E***, ***F***, and ***G*** represent a ratio of 1 representing no change in feature value between successive syllables. Results of statistics in Extended Data Figure 2-1.

10.1523/ENEURO.0199-25.2026.f2-1 Figure 2-1 Statistical test results for data from Fig 2. - Fig. 7 Download Figure 2-1, XLS file.

Previous studies have shown age-related changes, in song stereotypy and song speed, that are maximal in the first year post-hatch (Brainard and Doupe, 2001; Pytte et al., 2007; Glaze and Troyer, 2013; James and Sakata, 2019). In our dataset, we did not see a significant improvement in song similarity over age for the first songs of each bout (Fig. 3*A*; *p* = 0.4936, Kruskal–Wallis test). Since earlier studies have compared all songs within a bout (Pytte et al., 2007), we also repeated our analysis for songs within a bout but did not see any significant difference (Extended Data Fig. 3-1*C*; *p* = 0.494, Kruskal–Wallis test) possibly because of age differences with earlier studies. We did find differences in song temporal similarity in the first year post-hatch, i.e., when examining long-term changes in young adults (Extended Data Fig. 3-1*B*; *p* = 0.026, Kruskal–Wallis test). Given that song temporal similarity measures the correlation between motif temporal patterns, this is likely to be a direct result of changes in motif duration in the first year post-hatch. Similar to earlier studies (Brainard and Doupe, 2001; Pytte et al., 2007; Glaze and Troyer, 2013; James and Sakata, 2019), average first song duration reduced significantly only for long-term changes in young adults (Fig. 3*B*; *p* = 0.017, Kruskal–Wallis test). As shown above, mean IN number increased by 0.58 ± 0.07 (mean ± SE, Fig. 3*C*; *p* = 0.02, Kruskal–Wallis test, Tukey–Kramer test) and the associated “time-to-song” also increased by 119.74 ± 7.52 ms (mean ± SE, Fig. 3*D*; *p* = 0.00077, Kruskal–Wallis test), only for long-term changes in young adults. We next asked whether IN timing also reduced, similar to the reduction in song timing. Surprisingly, we found an increase in the interval between the first two INs (Fig. 3*E*; *p* = 0.009, Kruskal–Wallis test), while the interval between the last IN and song remained unchanged (Fig. 3*F*; *p* = 0.78, Kruskal–Wallis test). IN temporal similarity did not change significantly (Extended Data Fig. 3-1*A*).

10.1523/ENEURO.0199-25.2026.f3-1 Figure 3-1 (A - C) Changes in IN temporal similarity (A), motif temporal similarity (B) and within bout motif similarity (C). (D-I) Changes in IN acoustic properties, duration (D), mean frequency (E), entropy (F), log amplitude (G), pitch goodness (H) and frequency modulation (I). (J-O) Changes in song acoustic properties, duration (J), mean frequency (K), entropy (L), log amplitude (M), pitch goodness (N) and frequency modulation (O) are plotted across different age categories. Circles represent change in mean property between pairs of days in a particular age category for individual birds. Square and whiskers represent median and interquartile range for the 3 age groups. * denotes *p* ≤ 0.05 Kruskal Wallis ANOVA followed by Tukey-Kramer test. (see results of statistics in Fig. 2-1) Download Figure 3-1, TIF file.

We also saw small changes in acoustic features of songs (Extended Data Fig. 3-1*J–O*) and INs (Extended Data Fig. 3-1*D–I*) across days. However, given that it is difficult to ensure that the recording conditions are the same across different days (sometimes separated by >6 months), further controlled studies will be required to check the validity of these changes. Overall, these results showed that IN timing and number change only in the first year of age and remained stable after that period. Further, contrary to the reduction in song timing, our results showed that IN timing increased with age.

### IN features change in the direction of upcoming song

We next compared IN features with features of the first song syllable. As mentioned above, we were particularly interested in understanding how changes in IN features related to the features of the first song syllable. We chose the first recording day for each bird for this analysis for two reasons, namely, (1) we wanted maximum variability for our correlation analysis and, (2) the age range for the first recording day is smaller (90–120 d), thereby minimizing age-related effects. INs are characterized by three properties: the number of INs before song at the start of each bout, their timing measured by the intervals between successive INs, and their acoustic properties. The number of INs varies from bout to bout, and earlier studies have shown that the last IN represents a consistent state across bouts (Rajan and Doupe, 2013). Therefore, the variable number of INs has been hypothesized to ensure a consistent, final state is reached, despite variable first INs (Rajan and Doupe, 2013), and so we did not expect number of INs to correlate with any feature of song. As shown earlier (Rajan and Doupe, 2013; Rao et al., 2019), both the timing and the acoustic properties of INs changed systematically from the first to the last IN. While previous studies have shown changes in IN properties at the start of a bout (Rajan and Doupe, 2013; Rao et al., 2019), we extended these analyses by comparing IN properties to those of the first song syllable (Fig. 4; see Materials and Methods). For the acoustic properties, we focused on the mean and variability of individual features, rather than using a distance measure, to better understand what features of INs change during the progression from INs to song.

As INs progressed to song, significant changes in mean IN properties were in the direction of changes from INs to song (Fig. 4; repeated-measures one-way ANOVA followed by post hoc Tukey–Kramer test); by the last IN, some properties became similar to the first song syllable while other properties continued to change from the last IN to the first song syllable. Specifically, the mean intersyllable intervals reduced significantly from the first IN to the last IN (Fig. 4*A*; *p* = 0.0002) and was then comparable to the interval between the first two song syllables (Fig. 4*A*; *p* = 0.14). From the first IN to the last IN, INs became progressively longer (Fig. 4*B*; *p* = 0.02) and louder (Fig. 4*E*; *p* < 0.001). The first song syllable was longer (Fig. 4*B*; *p* = 0.002) and louder (Fig. 4*E*; *p* = 0.02) than the last IN. Mean frequency also increased from first IN to last IN to first song syllable, but this increase was not significant (Fig. 4*C*; *p* = 0.068). In contrast with changes in means from first to last IN, we found that the variability of all features were not significantly different between first IN and last IN (Fig. 4, column on the right; *p* > 0.05). From the last IN to the first song syllable, we found a significant reduction in the variability of entropy (Fig. 4*D*; *p* < 0.001). Previous studies have shown a reduction in the variability of inter-IN gaps, but in our results this trend was not significant (Fig. 4*A*, right; *p* = 0.29) possibly because of larger variability across birds. Overall, as INs progressed to song, these results replicated earlier results demonstrating changes in IN features. In addition, these results showed that IN timing and acoustic properties changed in the direction of the first song syllable with the last IN timing, duration, and amplitude becoming more similar to that of the first song syllable.

### IN acoustic features and not IN timing are correlated with corresponding first song syllable features

Previous studies have shown that similar last INs are followed by similar first song syllables (Rajan and Doupe, 2013). However, three important caveats necessitated a further examination of this correlation. First, similarity was measured using Euclidean distance in a four-dimensional space made up of four syllable acoustic features. This does not allow for a precise understanding of the actual features that contribute to the correlation. Second, as mentioned earlier, INs are also vocalizations like song, and it is unclear whether similar correlations exist between first INs and the first song syllable and between the first and second song syllables. Existence of such correlations would suggest a more global correlation between vocalizations belonging to a bout. Finally, vocalizations were recorded with a suspended microphone and this method of recording could also influence correlations as the position of the bird relative to the microphone need not be fixed for all bouts and the recording environment could also influence sound propagation.

To address the first two issues, we correlated first song syllable timing and acoustic properties with those of the first IN, the last IN, and the second song syllable (see Fig. 5 for an example of correlations). We found significant correlations in the timing of the first song syllable and the timing of the last IN in only 2/11 birds (Fig. 6*A*, median correlation strength, 0.16). None of the birds had significant correlations between the first song syllable timing, and the first IN timing and 4/11 birds showed significant correlations between first and second song syllable timing (Fig. 6*A*). Thus, a majority of birds did not show significant correlations between first song syllable timing and IN timing or second song syllable timing suggesting that the timing of all syllables are independent of each other.

10.1523/ENEURO.0199-25.2026.f6-1 Figure 6-1 (A, B) IN log amplitude for the First IN, Last IN and second song syllable were correlated with log amplitude of the first song syllable across bouts in birds with head-implanted microphones (A) and tracheosyringeal nerve cut birds (B). (C) Expiratory mean amplitude for the First IN, Last IN and second song syllable were correlated with the expiratory mean amplitude of the second song syllable across bouts in air-sac cannulated birds. Each circle represents r-values for within bout correlations (Pearsons correlation) in one bird. Lines connecting circles represent data from the same bird. Circles with a '*' represent significant correlations and unfilled circles represent non-significant correlations. Proportion of birds with significant correlations are indicated on top and marked with red if proportions were ≥ 0.5. * denotes p ≤ 0.05, ** denotes p ≤ 0.01, Wilcoxon signed-rank test. (see results of statistics in Fig. 2-1). Download Figure 6-1, TIF file.

10.1523/ENEURO.0199-25.2026.f6-2 Figure 6-2 Timing and acoustic properties of first song syllable (see Row Names) were correlated to the corresponding timing and acoustic properties for first IN position, last IN position or second song syllable (see Column names). Pearson correlation coefficient was calculated after removing outliers for one session in each bird and considered significant if p ≤ 0.05. Each cell indicates the proportion of birds with significant correlations (first row), the median R-squared value (middle row) and range of R-squared values for significant correlations (bottom row). Data reported here is for (1) birds implanted with a microphone on their head, and (2) birds with bilateral tracheosyringeal nerve lesions. This data supplements data shown in Fig. 6 and Fig. 6-1. Download Figure 6-2, DOCX file.

Contrary to the results with song timing, we found correlations between acoustic features in most birds. Specifically, log amplitude and mean frequency of the first song syllable were correlated with the corresponding features of the last IN in >50% of the birds (Fig. 6*C*,*E*; 8/13 birds for mean frequency and 13/13 birds for log amplitude). Surprisingly, we also found significant correlations between the acoustic features of the first song syllable and the first IN in most of these birds (Fig. 6*C*,*E*; 7/13 birds for mean frequency and 12/13 birds for log amplitude), albeit the strength of the correlations were slightly lower when compared with the strength of correlations between the last IN and the first song syllable (Fig. 6*C*,*E*; *p* = 0.09, mean frequency and *p* = 0.04, log amplitude for comparisons of *r* values, Wilcoxon signed-rank test). In addition, we also found correlations, of comparable magnitude, in mean frequency and log amplitude of the first and second song syllable (Fig. 6*C*,*E*).

As mentioned above, such correlations could also arise because of the recording conditions. To rule out this possibility, we examined similar correlations in two different datasets from different sets of birds. First, we analyzed acoustic recordings from birds with head-implanted microphones, where the microphone was always at a fixed distance from the bird (Suri and Rajan, 2018). A head-implanted microphone ensures constant distance between the bird and the microphone, but other aspects of the recording environment can still influence amplitude measurements through their effects on sound propagation. To control for this, we also analyzed respiratory pressure recordings (Kizhakoot et al., 2025) as an indirect proxy for amplitude as respiratory pressure is positively correlated with syllable amplitude (Plummer and Goller, 2008; Zollinger et al., 2011). These respiratory pressure recordings provided a measure of amplitude independent of the recording environment. In both sets of birds, we found similar correlations in log amplitude between the first song syllable and last IN and the first and second song syllables in 43% of the birds with head-implanted microphones (Extended Data Fig. 6-1) and 60% of the birds with respiratory pressure measurements (Extended Data Fig. 6-1). Correlations between the first song syllable and first IN were also present in a similar number of birds and were comparable in correlation strength. These results confirmed that most of the correlations in acoustic properties were not an artifact of our acoustic recording procedures. These data suggest the presence of global correlations in amplitude and mean frequency of syllables belonging to a bout.

To further examine the possibility of global correlations between syllables belonging to a bout, we examined correlations in birds with surgical lesions of the tracheosyringeal (ts) nerve. In birds with ts-nerve lesions, the brain no longer controls the syringeal muscles and so all syllables are reduced to harmonic stacks with the duration being similar to the prelesion syllable duration (Bottjer and Arnold, 1984; Vicario, 1991; Williams and McKibben, 1992; Roy and Mooney, 2007). Thus, syllable identity is reduced to only duration of the syllable as this is achieved through neural control of respiration. We found significant correlations in log amplitude in all birds with ts-nerve lesions (Extended Data Fig. 6-1) supporting the presence of global correlations between syllables belonging to a bout. In fact, we found correlations in other features as well suggesting a possible respiratory origin for these correlations.

Thus, while both IN timing and acoustic features change in the direction of song, only acoustic features (specifically, mean frequency and log amplitude) of the last IN, the first IN, and the second song syllable were significantly correlated with those of the first song syllable suggesting the presence of global correlations in log amplitude and mean frequency of syllables belonging to a bout.

### IN acoustic features share similarities with other syllable repeats but speeding up of intervals between INs is unique to IN repeats

INs are also vocalizations like song but one important difference between song syllables and INs is repetition; while song syllables typically do not repeat, INs repeat before progressing to song. To examine whether changes in timing and acoustic features of INs could be a feature of all vocalizations that repeat, we compared the timing and acoustic features of INs with those other vocalizations that also repeat. Song syllable repeats were present in a small fraction of zebra finches (Fig. 7*A*, top; example spectrogram with repeat of the song syllable “*c*”) and zebra finches also repeat calls (other non-song vocalizations that can be produced outside of song bouts for communication) outside of their song bouts (Fig. 7*A*, bottom). To compare repeats across these three categories (INs, song syllable repeats, and call repeats), we chose birds with song syllable repeats and compared the properties of song syllable repeats with those of IN repeats and call repeats (if present) from the same birds. Mean number within a repeat for song syllable repeats was significantly smaller compared with mean IN number (Fig. 7*B*; *p* = 0.02, Kruskal–Wallis test followed by Tukey–Kramer test), while mean repeat number for calls was not different from mean IN number (Fig. 7*B*; *p* = 0.81, Kruskal–Wallis test). Variability of repeat number was similar across all categories (Fig. 7*C*; *p* = 0.19, Kruskal–Wallis test).

The intervals between repeat syllables showed interesting differences between the three categories of repeats. For all categories of repeats, we compared the mean interval between the first two syllables in the repeat sequence (first interval) and the last syllable of the repeat sequence and the next syllable in the bout (last interval). As shown earlier (Rajan and Doupe, 2013; Rao et al., 2019), for IN repeats, the last interval was significantly shorter than the first interval (Fig. 7*D*), while the opposite was true for call repeats with the last interval being significantly longer than the first interval (Fig. 7*D*). Interestingly, for song syllable repeats, we observed differences depending on the position of the repeat within the song motif; for repeats that were the last syllable of the song, the last interval was longer than the first interval, though this difference was not statistically significant (Fig. 7*D*), while for repeats that occurred at the beginning of the song, the last and first interval were not significantly different (Fig. 7*D*). Thus, the speeding up of intervals between successive INs was a unique feature of INs (Fig. 7*E*; ratio of successive intervals is <1 only for IN repeats; mean ± SE, 0.66 ± 0.03).

To compare acoustic features, we focused on duration and amplitude of the first and last syllable of the repeat, as these features changed significantly for INs (Fig. 4). As we were interested in changes in feature values during a repeat, we focused on the ratio of feature values between successive syllables of the repeat. In this set of birds, ratios were not significantly different between repeat types (Fig. 7*E*, *p* = 0.648, Kruskal–Wallis test and Fig. 7*G*, *p* = 0.956, Kruskal–Wallis test). We did not see significant changes for INs too, possibly due to greater variability in individual birds. Overall, these results showed that average repeat number for INs was more than repeat number for song syllable repeats. While the timing of most syllable repeats changed, the speeding up of gaps between successive syllables of the repeat was unique to INs.

## Discussion

INs are short, repeated vocalizations that occur at the beginning of a male zebra finch's song bout. Due to their similarity to neural preparatory activity, INs have been proposed to reflect motor preparation for the complex song sequence. However, unlike neural preparatory activity that occurs before any movement, INs are also vocalizations/movements, like the song that follows, suggesting the possibility that INs are part of the song sequence. Here, as a first step to determining this, we compared the properties of INs and song syllables. First, we found that mean IN number increased by 0.58 ± 0.07 in the first year post-hatch. In addition to this increase, song duration reduced but the silent interval between the first two INs increased. Second, from first IN to last IN, we found that the timing, duration, and amplitude of INs became more similar to that of the first song syllable. Third, IN timing was correlated with song timing in only 18% of birds. In contrast, both first and last IN acoustic properties, specifically amplitude and mean frequency, were correlated with that of the first song syllable, in >40% of birds. In addition, the first and second song syllables were also correlated in these birds. Finally, when compared with other repeated vocalization, the timing of INs followed a different pattern of change.

Overall, the similarities in acoustic properties between INs and song syllables suggest that INs are part of the song sequence. While the timing of INs becomes more song-like, the lack of correlations between IN timing and song timing and difference in IN timing when compared with other syllable repeats suggest that IN timing is controlled by other mechanisms.

### Age-related changes in IN properties

INs represent the variable portion of song bouts. In our dataset, we found significant increases in the gap between the first two INs and in mean IN number, in the first year after hatching. The increase in mean IN number could simply be a result of a significant increase in the gap between the first two INs, as previous studies have shown that longer gaps between the first two INs are correlated with more number of INs at the start of individual bouts (Rajan and Doupe, 2013; Rao et al., 2019). Beyond the first year of age, we did not find any changes in IN properties. The timeline of these changes parallels previously documented changes to song with larger changes in the first year of age, followed by greater stability after the first year of age (Brainard and Doupe, 2001; Kao and Brainard, 2006; Pytte et al., 2007; Glaze and Troyer, 2013; James and Sakata, 2019). Similar timelines for age-related changes in INs and song suggest the possibility for a common underlying mechanism that triggers changes restricted to this time period. Further, the gap between the first two INs increased while song duration reduced, suggesting differential control of these two aspects.

### Last IN timing and acoustic properties are more similar to those of the first song syllable

Our results show that as INs progress to song, the timing and acoustic properties (specifically duration and amplitude) of the last IN were more similar to that of the first song syllable. This shows that IN properties change to become more like those of song, suggesting an increasing involvement of the song control pathway in controlling IN timing and acoustic features. While there is no direct evidence for this, there are other pieces of evidence that partially support this idea and suggest further experiments. First, cooling of premotor nucleus HVC only stretches INs to 70% of the extent of stretching seen in song syllables (Long and Fee, 2008). If there is greater involvement of the song control pathway during the last IN compared with the first IN, one would expect the last IN stretch, upon cooling, to be similar to the stretch of song syllables, while first INs would be expected to stretch to a smaller extent. Future cooling experiments in HVC could carefully examine differences in the extent of stretching of first and last INs. Second, disruption of auditory (Naie and Hahnloser, 2011) or thalamic (Coleman and Vu, 2005; Elmaleh et al., 2021) or dopaminergic inputs (Ben-Tov et al., 2023) to premotor nucleus HVC result in birds producing long strings of INs (during female-directed courtship song) that either progress to song less frequently or do not progress to song at all. Lesions of A11, the nucleus that provides dopaminergic inputs to HVC, completely abolish IN and courtship song production, when presented with a female (Ben-Tov et al., 2023). These inputs are thought to provide an excitatory drive to HVC (Otchy et al., 2015) and consequently loss of this excitatory drive might affect IN progression to song. Thus, our results showing that IN features become more similar to song, as INs progress from first to last IN, suggest an increasing involvement of HVC in production of INs.

### Amplitude and mean frequency of INs are correlated with those of the first song syllable in >40% of birds

Our results show correlations in the log amplitude and mean frequency of the last IN and the first song syllable. While this is expected from the motor preparation hypothesis, we also found correlations between the first and second song syllables and the first IN and first song syllable, suggesting the presence of global correlations between syllables within a bout. Similar correlations in the duration of syllables within a song motif have been described earlier (Glaze and Troyer, 2006). Our results showing that such correlations extend to INs as well suggest that IN properties might reflect the fact that they are also syllables similar to song syllables, possibly because of shared neural control. What could drive such global correlations? Neural activity of individual neurons in motor nucleus RA, downstream of HVC, has been shown to be correlated with variations in mean frequency and amplitude of individual syllables (Sober et al., 2008). Our results showing the presence of global correlations within a bout would suggest correlated activity of neurons across different syllables of a bout.

Interestingly, IN timing was correlated with song syllable timing in only 18% of the birds. This suggests that IN timing does not share similarities with song timing. This result is consistent with previous analyses (Glaze and Troyer, 2006); experimental cooling of HVC (Long and Fee, 2008; Andalman et al., 2011) has shown that syllables and gaps differ in their correlations and responses to HVC cooling. While syllables are thought to be driven by chains of neurons within HVC, gaps, corresponding to inhalations, may be driven by brainstem respiratory neurons (Andalman et al., 2011; Goller, 2025). Such differential control of syllables and gaps could explain our results showing fewer correlations between IN timing and song timing.

### Are INs similar to other repeating syllables?

INs are different from other song syllables because INs repeat, while other song syllables, typically, do not repeat. However, some zebra finches repeat song syllables (Helekar et al., 2003) and many zebra finches repeat call syllables (Zann, 1996; non-song syllables that can be produced independently and are used for communication). Across these three categories of repeats (IN repeats, song syllable repeats, and call repeats), we found differences in the gap between successive syllables of a repeat. For INs, the gap between successive INs reduces, while for calls and song syllables at the end of the motif, we found that gaps between successive syllables increased, though the increase was not significant for motif syllables at the end (Fig. 7*D*). In Bengalese finches, a songbird species with a variable song sequence, earlier studies have shown that the gap at a branchpoint syllable (a syllable that can be followed by any one of several different syllable types) is inversely proportional to the probability of the transition, i.e., shorter gaps are associated with more probable transitions, while longer gaps are associated with less probable transitions (Matheson and Sakata, 2015; Tachibana et al., 2015; Troyer et al., 2017). It is possible that the gap between the last IN and song follows this as the transition to song is a prominent transition from the last IN. Repeat syllables are common in Bengalese finches (Okanoya, 2004), and previous studies have shown that repeat number is sensitive to perturbation of auditory feedback (Wittenbach et al., 2015). INs in the zebra finch are not affected immediately after deafening (Rao et al., 2019), suggesting the possibility that INs differ from other repeats. Further analysis of repeats across different songbird species might provide insights into whether INs are similar to other repeat syllables found in other songbird species. A comparative analysis of whether other songbird species have INs could also provide more information on the origin and function of INs and their relation to other types of repeating syllables.

### What do INs reflect?

As mentioned above, INs have been hypothesized to represent motor preparation due to similarities with neural preparatory activity seen in primates and rodents, before the start of simple movements. However, unlike neural preparatory activity that occurs before any movement, INs are vocalizations/movements like the song that follows. Further, recent results have shown the presence of preparatory activity, in HVC, before the first IN of a song bout (Rajan, 2018; Daliparthi et al., 2019). As discussed above, many of our results suggest that the change in IN properties from first to last IN reflects a slow change in control of vocalizations with HVC (and other song control nuclei) gradually taking over control of vocalizations, and this could represent a continuation of the preparatory activity that started in HVC before the first IN. The gradual take-over in HVC could result in greater activity in RA and could lead to more song-like timing and acoustic properties in successive INs. Once started, coordinated activity in RA could drive correlations within all syllables of a bout. Further experiments involving optogenetically driven, temporally controlled, disruption of activity, in HVC or RA projection neurons, during INs could help distinguish this possibility. Overall, our results suggest that changes in the acoustic properties of INs reflect the fact that INs are also vocalizations like song, while changes in the timing of INs reflect differences with song and potentially, different mechanisms for controlling timing.

## Synthesis

Reviewing Editor: Charlotte Cornil, Universite de Liege Decisions are customarily a result of the Reviewing Editor and the peer reviewers coming together and discussing their recommendations until a consensus is reached. When revisions are invited, a fact-based synthesis statement explaining their decision and outlining what is needed to prepare a revision will be listed below. The following reviewer(s) agreed to reveal their identity: Todd Troyer.

The authors have significantly streamlined and focused the flow of arguments in the manuscript resulting in an improved manuscript. Even though the advances are mostly incremental it is now much clearer how the results extend previous work. However, there are several places in the text and figures that need more work, chiefly to improve the clarity of the presentation. In particular, there are still places where the motivation to conduct a specific comparison and which question it is supposed to inform on and how remains unclear. It would be helpful for the reader to have these assumptions/hypotheses and expectations spelled out and related to the specific analysis.

We are providing examples here but the authors are asked to carefully check the manuscript for all potential lack of clarity.

Abstract: While framing the work in the context of preparatory neural activity is OK, the relationships between this work and previous studies related to neural preparation are subtle and there aren't really any contrasts tested in this manuscript. Since we do not see any important way that the paper would be changed if one simply assumed that the acoustic parameters were a good read out of the underlying neural representation, we suggest adding a background sentence before the main results. These include: the replication of the results that INs approach an end state before advancing to the song motif, the stronger correlation with acoustic over timing parameters between INs and song, the fact that IN repeats act differently than other repeats, and the data on regularity over development. Moreover, the analysis of changes in INs over age still represent a significant proportion of the results and contribute to the conclusions, but are not mentioned at all in the abstract and significance statement. These results should be succinctly described and summarized in the abstract.

Introduction:

The last paragraph in the Introduction (lines 60-86) is hard to parse. Several different issues were discussed and it was often unclear exactly what the specific question was that the authors intended to address. Perhaps the authors can break this down into a couple of paragraphs, making each part clearer, more like the writing in the Discussion section.

Methods and results

Another example is the argument that if INs serve preparatory purposes, they should get more similar to song. It is assumed this is because preparatory neural activity has been shown to get more similar to the actual activity during the movement. If so, it would be helpful for the reader to have these assumptions/hypotheses and expectations spelled out and related to the specific analysis.

There is also the question whether mean frequency is an amplitude related measure. Yes, it is the frequency with the highest amplitude in the frequency spectrum, but that does not change by increasing the amplitude of a vocalization (which is how the sentence is understood).

Figures are often confusing. The main problems are listed here along with suggestions for improvement. These are merely suggestions not prescriptions, but the authors are urged to address the problem raised in some way.

Figure 2

In 2A, it is hard to see which line belongs to which dot. Moreover, it is said that red indicates significant increases and black represents significant decreases. But in some cases, the data at least looks exactly the opposite way. Do these relate to birds with multiple comparisons? Which comparison is used to describe significant increases/decreases?

Fig 2B, is a bit hard to interpret. Many circles seem to have the same or very similar color. Additionally, because of how the data is presented, the lower right half of the plot is unused. This mostly shows the ages at comparison, but it is very hard to represent quantities with circle size and most of the circles look the same (presumably small differences). It looks like nearly all comparisons near the same age give significant differences in the first year. This might be because IN # is variable in the first year. But then there are many non-significant differences when comparing year 1 to year 2/3. Where you see nearly all differences being significant is comparing year 2 to year 3. Could it be an option to change it a bit to represent age 1 on the x-axis, but change the y-axis to the distance between age 1 and 2 and encode the change as color of the dot instead of size? This might emphasize the point the authors want to make in a clearer way. Alternatively, a version of 2A where the colors were clear might be OK.

Figure3

- It is hard to figure what the tick label '<1 yr-> 1 yr' means. Maybe using vs. instead of ->?

- Is there a bias between the three groups in the time span that is included? In other words, could changes be bigger in the middle group because on average more time passed between two points than in the other two groups?

- Aren't the changes in 3D and E a consequence of an increase in IN number (and the fact that the last IN seems to be some final state)?

Figure 5.

- there seems to be a mixup of the data and the labels. If first song syllable is on the y-axis in all plots, the range of values should not change.

- Why does C use blue dots?

Figure 6. Given that one of the biggest worries about these correlations is the possibility of bias due to recording conditions, perhaps you can use a different color or icon (circle vs. square?) for recording using a clipped microphone vs. head-fixed. We guessed blue means second syllable. Is this correct? This needs to be mentioned in the legend or just eliminated.

Fig. 7. More clarity on the definition of ratio and that it applies to E-G is needed. Maybe use 'first/last ratio' to label each of the vertical axes and then edit legend to say something more specific. The legend says successive syllables. What about repeats with 3 or more? Is this really a first vs. last? Please clarify.

Table. The numbers would be much easier to read if you truncated at three significant digits. Also separating the fraction, mean r, and range of r could be written on separate lines instead of relying on automated

## Author Response

Response to Reviewers Synthesis Statement for Author (Required):

The authors have significantly streamlined and focused the flow of arguments in the manuscript resulting in an improved manuscript. Even though the advances are mostly incremental it is now much clearer how the results extend previous work. However, there are several places in the text and figures that need more work, chiefly to improve the clarity of the presentation. In particular, there are still places where the motivation to conduct a specific comparison and which question it is supposed to inform on and how remains unclear. It would be helpful for the reader to have these assumptions/hypotheses and expectations spelled out and related to the specific analysis.

We are providing examples here but the authors are asked to carefully check the manuscript for all potential lack of clarity.

We thank the editor and the reviewers for their thoughtful comments and their constructive feedback on the revised manuscript. We have addressed all of the concerns and we feel that the manuscript has further improved significantly.

Below, we have provided details of how we have addressed the comments. Reviewer comments are in black and our responses are in blue. Portions that have been copied directly from the revised manuscript are in red and the line numbers from the article file are provided.

Abstract: While framing the work in the context of preparatory neural activity is OK, the relationships between this work and previous studies related to neural preparation are subtle and there aren't really any contrasts tested in this manuscript. Since we do not see any important way that the paper would be changed if one simply assumed that the acoustic parameters were a good read out of the underlying neural representation, we suggest adding a background sentence before the main results. These include: the replication of the results that INs approach an end state before advancing to the song motif, the stronger correlation with acoustic over timing parameters between INs and song, the fact that IN repeats act differently than other repeats, and the data on regularity over development. Moreover, the analysis of changes in INs over age still represent a significant proportion of the results and contribute to the conclusions, but are not mentioned at all in the abstract and significance statement. These results should be succinctly described and summarized in the abstract.

We thank the reviewers and the editor for these suggestions. We have worked on these and have revised the abstract. The revised abstract is pasted below. "Male zebra finches begin their song bouts by repeating a short sound called an introductory note (IN). As INs repeat, their timing and acoustic properties change, similar to pre-movement neural preparatory activity seen in primates and rodents. Hence, INs are thought to reflect motor preparation for starting song. However, INs themselves are vocalizations/movements, like the song that follows, suggesting that INs could be part of the song sequence. To determine how similar songs and INs are, we compared their properties in adult, male zebra finches recorded across 3 years. First, song and INs simultaneously changed in the first year post-hatch; as described earlier, songs became faster and we found an increase of 0.58 +/- 0.07 in mean IN number. Second, as described earlier, IN properties changed from first to last IN. Here, we found that these changes were driven by the last IN becoming more like the first song syllable in duration, amplitude and timing. Third, within each bout, first song syllable timing was correlated with last IN timing in only 18% of birds. Amplitude and mean frequency of the first song syllable was correlated not only with the last IN, but also with the first IN and second song syllable in > 40% of birds. Finally, the reduction in gaps between successive INs was not seen in other types of repeat syllables. Overall, the acoustic similarities between INs and song suggest that INs are part of the song sequence, but timing differences suggest different mechanisms for controlling IN timing." Line Nos: 2-18 in the revised manuscript Introduction:

The last paragraph in the Introduction (lines 60-86) is hard to parse. Several different issues were discussed and it was often unclear exactly what the specific question was that the authors intended to address. Perhaps the authors can break this down into a couple of paragraphs, making each part clearer, more like the writing in the Discussion section.

We thank the reviewers and the editor for this suggestion. We have broken down the different issues into paragraphs. The modified introduction is pasted below. "Simple movements, like reaching for an object, are preceded by a "preparatory" period (Fig. 1A, left) when neural activity progresses from a variable initial state to a consistent final state just before movement initiation (Fig. 1A, right) (Churchland et al., 2006b, 2010; Shenoy et al., 2011; Li et al., 2016; Svoboda and Li, 2018). Recent studies suggest that preparation also precedes complex, naturally learned, movement sequences, like the well-studied song of the adult male zebra finch (Rajan and Doupe, 2013; Rao et al., 2019), but the nature of this preparation remains poorly understood.

Zebra finch song is a stereotyped sequence of sounds interleaved by silent gaps (Fig. 1B, left, 'a' and 'b' represent the song sequence). It is part of the courtship ritual of the male zebra finch (Sossinka and Böhner, 1980; Zann, 1996; Fee and Scharff, 2010) and is naturally learned by young birds (Immelmann, 1969; Price, 1979; Zann, 1996). Song bouts begin with variable repetitions of a short sound called an introductory note (IN, marked as 'i' in Fig. 1B) (Price, 1979; Sossinka and Böhner, 1980). As INs repeat, their timing and acoustic features change; gaps between successive INs reduce and become more stereotyped, and acoustic features reach a consistent last IN just before the start of the first song (Fig. 1B, right) (Rajan and Doupe, 2013; Rao et al., 2019). This progression resembles neural preparatory activity going from a variable, initial state to a consistent, final state before movement initiation. Neural preparatory activity has been extensively characterized in rodent and primate brains and is believed to represent motor preparation for initiation of simple movements (Shenoy et al., 2011, 2013; Churchland, 2015; Svoboda and Li, 2018). By analogy, INs might also reflect motor preparation for complex, naturally learned song sequences.

In addition to similarities in progression to movement, INs and neural preparatory activity both correlate with features of the upcoming movement. In primates, pre-movement neural activity correlates with upcoming movement parameters on a trial-by-trial basis in a subset of neurons (Churchland et al., 2006a). In zebra finches similar last INs are followed by similar first song syllables (Rajan and Doupe, 2013). This adds support to the hypothesis that INs reflect motor preparation for initiating the complex song sequence.

Despite these similarities one important difference remains. Preparatory neural activity occurs before the movement and is itself not accompanied by movement. In contrast, INs are movements (vocalizations) like the song that follows. Additionally, neurons in multiple song system nuclei, in the zebra finch brain, show activity changes before the first IN, when the bird is not vocalizing (Hessler and Doupe, 1999; Kao et al., 2008; Woolley et al., 2014; Rajan, 2018; Daliparthi et al., 2019). Recent studies suggest this activity reflects preparation for song initiation (Rajan, 2018; Daliparthi et al., 2019). Then what do INs represent? INs could either reflect a "vocalized" continuation of preparatory activity or they could reflect variable vocalizations that are part of the song sequence, similar to variability seen in the song sequences of related songbird species, like the Bengalese finch (Okanoya, 2004). To address these possibilities we recorded and analyzed INs and songs from multiple adult, male zebra finches.

Specifically, we first characterized age-related changes in INs. Song is known to speed up and become more stereotyped over the first year of age (Brainard and Doupe, 2001; Pytte et al., 2007; Glaze and Troyer, 2013; James and Sakata, 2019). If INs are part of the song sequence, IN features should also show similar age-related changes. Previous studies have shown that IN features do not change over a 5 day period (Rao et al., 2019), but longer term changes remain unclear. Here, we characterized IN changes over the first 3 years post-hatch through repeated recordings of individual birds.

Second, we compared IN features with first song syllable features. While IN timing and acoustic properties change from first to last IN and similar last INs precede similar first song syllables (Rajan and Doupe, 2013),what features drive these changes and whether they are in the direction of song remains unclear. While neural preparatory activity is correlated with upcoming movement parameters, the neural preparatory hypothesis does not make specific claims about correlations with actual movement related activity; preparatory activity is hypothesized to set the initial state for movement dynamics to unfold without necessarily being in the direction of movement related activity (Churchland et al., 2010; Shenoy et al., 2011, 2013). However, if INs are not preparatory, but are part of the song sequence, one would expect IN features to change and become closer to song features. Additionally, just like correlations between the last IN and the first song syllable, one would also expect similar correlations between the first IN and the first song syllable and between the first and second song syllables. Presence of such correlations would suggest the existence of global correlations between vocalizations within a song bout and be consistent with the idea of INs being part of the song sequence.

Finally, unlike typical zebra finch songs that consist of distinct, non-repeating syllables, INs involve repeating the same syllable. This raises the question of whether IN properties reflect general properties of vocalizations that repeat. Songs of a small fraction of zebra finches do contain repeats of song syllables and non-song vocalizations (Helekar et al., 2003). Therefore, we also compared IN properties with those of other repeating syllables to determine whether IN properties reflect general properties of repeating syllables. "

Line Nos: 44 - 116 in the revised manuscript Methods and results Another example is the argument that if INs serve preparatory purposes, they should get more similar to song. It is assumed this is because preparatory neural activity has been shown to get more similar to the actual activity during the movement. If so, it would be helpful for the reader to have these assumptions/hypotheses and expectations spelled out and related to the specific analysis.

We thank the reviewers and the editor for pointing this out and apologize for the lack of clarity. The neural preparatory hypothesis does not make very specific claims about whether INs should become similar to song. Infact, in primates, neural preparatory becomes similar to movement related activity only in some neurons. More generally, in primates, neural preparatory activity changes in an abstract dimension that is not necessarily related to movement activity. This has led to the idea that neural preparatory activity sets the initial state for the dynamics of movement related activity to unfold. We have added a few lines to explain this in the Introduction (pasted below). "Second, we compared IN features with first song syllable features. While IN timing and acoustic properties change from first to last IN and similar last INs precede similar first song syllables (Rajan and Doupe, 2013),what features drive these changes and whether they are in the direction of song remains unclear. While neural preparatory activity is correlated with upcoming movement parameters, the neural preparatory hypothesis does not make specific claims about correlations with actual movement related activity; preparatory activity is hypothesized to set the initial state for movement dynamics to unfold without necessarily being in the direction of movement related activity (Churchland et al., 2010; Shenoy et al., 2011, 2013). However, if INs are not preparatory, but are part of the song sequence, one would expect IN features to change and become closer to song features. Additionally, just like correlations between the last IN and the first song syllable, one would also expect similar correlations between the first IN and the first song syllable and between the first and second song syllables. Presence of such correlations would suggest the existence of global correlations between vocalizations within a song bout and be consistent with the idea of INs being part of the song sequence." Line Nos. 95 - 109 in the revised manuscript.

Where necessary, we have also added a line in each of the results sections to clearly mention the motivation for specific analyses.

There is also the question whether mean frequency is an amplitude related measure. Yes, it is the frequency with the highest amplitude in the frequency spectrum, but that does not change by increasing the amplitude of a vocalization (which is how the sentence is understood).

We agree with this and have removed the last line from the definition of mean frequency. The definition of mean frequency now reads: "Mean Frequency: The audio signal can be decomposed into different frequencies present in the signal. The mean frequency is a pitch measure that assesses the center of the distribution of intensity across different frequencies. It is measured in Hz." Line Nos: 295 - 297 in the revised manuscript Figures are often confusing. The main problems are listed here along with suggestions for improvement. These are merely suggestions not prescriptions, but the authors are urged to address the problem raised in some way.

Figure 2 In 2A, it is hard to see which line belongs to which dot. Moreover, it is said that red indicates significant increases and black represents significant decreases. But in some cases, the data at least looks exactly the opposite way. Do these relate to birds with multiple comparisons? Which comparison is used to describe significant increases/decreases? We apologize for the confusion. The red lines indicate cases where the difference between any two of the days is significant as per the Kruskal Wallis ANOVA and the largest change between any two days is positive. The change need not be consistently positive or negative throughout the time period. Similarly black lines represent cases where the difference between any two days is significant as per the Kruskal Wallis ANOVA and the largest change between any two days is negative. We have clarified this in the Results section.

Fig 2B, is a bit hard to interpret. Many circles seem to have the same or very similar color. Additionally, because of how the data is presented, the lower right half of the plot is unused. This mostly shows the ages at comparison, but it is very hard to represent quantities with circle size and most of the circles look the same (presumably small differences). It looks like nearly all comparisons near the same age give significant differences in the first year. This might be because IN # is variable in the first year. But then there are many non-significant differences when comparing year 1 to year 2/3. Where you see nearly all differences being significant is comparing year 2 to year 3. Could it be an option to change it a bit to represent age 1 on the x-axis, but change the y-axis to the distance between age 1 and 2 and encode the change as color of the dot instead of size? This might emphasize the point the authors want to make in a clearer way. Alternatively, a version of 2A where the colors were clear might be OK.

We apologize for the confusion and have now provided 3 new panels highlighting the different days selected for each of the different categories in Fig. 3. Fig. 2B, 2C and 2D are the new panels that show the selected days for each of the 3 categories. These panels make the point that we had intended to make; i.e. the largest changes between days are typically when we consider long-term changes in young adults, such that the first day considered is < 1 year and the second day considered is > 1 year (more red filled triangles in 2C compared to 2B and 2D).

Figure3 - It is hard to figure what the tick label '<1 yr-> 1 yr' means. Maybe using vs. instead of ->? We apologise for the confusion here. We have now changed the labels to "Young adult Day-to-day, Young adult Long-term, Old adult Long-term" as these reflect that actual changes that we are describing here. We hope this makes it clearer.

- Is there a bias between the three groups in the time span that is included? In other words, could changes be bigger in the middle group because on average more time passed between two points than in the other two groups? This is not the case for the birds. The difference in days is actually higher in the second group. Median and range for the different groups are Group 1 - 1 [1, 3] Group 2 - 507 [204, 640] Group 3 - 301.5 [108 711] Also shown below is a figure showing difference in days vs. mean IN difference for the 3 groups. If we just look at change in days, it is more in group 2 than group 3. However, within group there is no trend for higher change in mean related to more change in days. Each dot shows the difference between two days that were selected in each group. The gray square shows the mean across all points.

- Aren't the changes in 3D and E a consequence of an increase in IN number (and the fact that the last IN seems to be some final state)? Yes, 3D and E are related and complementary to the results showing an increase in IN number. Theoretically, mean IN number could increase without actual changes in the time-to-song. Here, we show that the increase in mean IN number is associated with an increase in the time to start song (from the first IN onset) and an increase in the silent interval between the first two INs. It is possible that 3E is the main cause for changes in 3C and 3D. Our previous results (Rajan and Doupe, 2013; Rao et. al., 2019) show that both mean IN number and time-to-song are positively correlated with the silent interval between the first two INs.

Figure 5.

- there seems to be a mixup of the data and the labels. If first song syllable is on the y-axis in all plots, the range of values should not change.

We thank the reviewers and the editor for spotting this and we apologize for this error. We have fixed this now. First song syllable was supposed to be the label for the x-axis and we have corrected this now.

- Why does C use blue dots? Blue dots indicate song syllable and this is consistent with Fig. 3, Fig. 4 and Fig. 6. The new figure legend mentions this as follows. "Fig. 5 Representative example of within bout correlations between acoustic properties of IN and song (A-C) Example session from one bird that shows significant positive correlations for Log Amplitude of first song syllable in the (first) motif along x-axis with (A) First IN position (black circles), (B) Last IN position (black circles) and (C) second song syllable (blue circles) respectively along y-axis. Circles represent data from individual bouts. p {less than or equal to} 0.05, Pearson correlation coefficient calculated after removing outliers. (Results of statistics in Fig. 2-1)." Line Nos. 811 - 818 in the revised manuscript.

Figure 6. Given that one of the biggest worries about these correlations is the possibility of bias due to recording conditions, perhaps you can use a different color or icon (circle vs. square?) for recording using a clipped microphone vs. head-fixed. We guessed blue means second syllable. Is this correct? This needs to be mentioned in the legend or just eliminated.

We apologize for the confusion. Yes, blue means song syllable and in this case second song syllable correlation with first song syllable. This has been mentioned in the legend now as follows. "Fig. 6 Correlations with first song syllable (A-G) IN timing (A) and acoustic properties (duration (B), mean frequency (C), entropy (D), log amplitude (E), pitch goodness (F) and frequency modulation (G) for a syllable) for the First IN, Last IN and second song syllable were correlated with corresponding properties of the first song syllable across bouts. Each circle represents r-values for within bout correlations (Pearsons correlation) in one bird. Lines connecting circles represent data from the same bird. Circles with a '*' represent significant correlations and unfilled circles represent non-significant correlations. Proportion of birds with significant correlations are indicated on top and marked with red if proportions were {greater than or equal to} 0.5. * denotes p {less than or equal to} 0.05, ** denotes p {less than or equal to} 0.01, Wilcoxon signed-rank test. (Results of statistics in Fig. 2-1)." Line Nos. 820 - 829 in the revised manuscript.

This figure only shows correlations for suspended microphone. Correlations for head-fixed microphone recordings, respiratory pressure recordings and ts-cut recordings are shown in Supp. Fig.6-1 and in Table 1.

Fig. 7. More clarity on the definition of ratio and that it applies to E-G is needed. Maybe use 'first/last ratio' to label each of the vertical axes and then edit legend to say something more specific. The legend says successive syllables. What about repeats with 3 or more? Is this really a first vs. last? Please clarify.

We apologize for the confusion. This is not first/last ratio. Ratios were calculated for all pairs of successive syllables. As for repeat length, for calculating mean and CV of repeat number all repeat lengths were included - a repeating syllable can have instances where it repeated only once. This was of course excluded for selecting instances of call repeats - otherwise the means would be highly skewed.

The filled circles indicate MEAN repeat number > 2.

For comparing first-last intervals and ratios, repeat instances less than 2 are ignored. However, the filled circles here still mark those repeats whose mean repeat numbers were >2.

Table. The numbers would be much easier to read if you truncated at three significant digits. Also separating the fraction, mean r, and range of r could be written on separate lines instead of relying on automated We apologize for making this complicated. We have made changes as suggested.
