# Supplementary material for: Zebra Finch Introductory Notes Become Song-Like But Have Different Timing and Acoustic Correlations with the Song That Follows
Source: eNeuro. 2026 Aug 7;13(8):ENEURO.0199-25.2026. doi: 10.1523/ENEURO.0199-25.2026 (PMC13471931; doi:10.1523/ENEURO.0199-25.2026)
Supplement: Supplementary file 1 [file eneuro-13-ENEURO.0199-25.2026-s004.docx]

|  |  | **Unmanipulated birds** | | | **Surgically manipulated birds** | | |
| --- | --- | --- | --- | --- | --- | --- | --- |
|  |  | **Head fixed microphone (n=6)** | | | **Ts Cut (n=6)** | | |
|  | **Song Feature** | First IN | Last IN | Second  Song syllable | First IN | Last IN | Second  Song syllable |
|  | Duration | 3/7  0.126  0.105 - 0.348 | 0/7 | 1/7  0.41 | 4/6  0.186  0.019 - 0.469 | 2/6  0.319  0.115 - 0.523 | 4/6  0.177  0.107 - 0.408 |
|  | Mean  Frequency | 0/7 | 2/7  0.283  0.261 - 0.305 | 3/7  0.215  0.181 - 0.232 | 6/6  0.116  0.071 - 0.534 | 6/6  0.274  0.086 - 0.675 | 5/6  0.242  0.141 - 0.602 |
|  | Entropy | 1/7  0.228 | 2/7  0.216  0.213 - 0.219 | 4/7  0.23  0.102 - 0.371 | 3/6  0.181  0.074 - 0.362 | 4/6  0.466  0.258 - 0.581 | 6/6  0.304  0.152 - 0.513 |
|  | Pitch  Goodness | 1/7  0.55 | 1/7  0.274 | 0/7 | 6/6  0.325  0.139 - 0.692 | 6/6  0.473  0.237 - 0.83 | 6/6  0.633  0.317 - 0.779 |
|  | Frequency  Modulation | 0/7 | 0/7 | 2/7  0.289  0.149 - 0.43 | 4/6  0.129  0.039 -0.163 | 3/6  0.243  0.22 - 0.365 | 4/6  0.221  0.182 - 0.499 |

**Fig. 6-2**

Timing and acoustic properties of first song syllable (see Row Names) were correlated to the corresponding timing and acoustic properties for first IN position, last IN position or second song syllable (see Column names). Pearson correlation coefficient was calculated after removing outliers for one session in each bird and considered significant if p ≤ 0.05. Each cell indicates the proportion of birds with significant correlations (first row), the median R-squared value (middle row) and range of R-squared values for significant correlations (bottom row). Data reported here is for (1) birds implanted with a microphone on their head, and (2) birds with bilateral tracheosyringeal nerve lesions. This data supplements data shown in Fig. 6 and Fig. 6-1.
